# An Efficient Evolutionary Neural Architecture Search Algorithm Without Training

**DOI:** 10.3390/biomimetics10070421

**Published:** 2025-06-29

**Authors:** Yang An, Changsheng Zhang, Jintao Shao, Yuxiao Yan, Baiqing Sun

**Affiliations:** 1 Software College, Northeastern University, Shenyang 110167, China; 2110502@stu.neu.edu.cn (Y.A.); 2110501@stu.neu.edu.cn (Y.Y.); 2290131@stu.neu.edu.cn (B.S.); 2 College of Computer Science and Engineering, Ningxia Institute of Science and Technology, Shizuishan 753000, China; 3 Lightspeed Studios, Tencent Co., Ltd., Shenzhen 518000, China; jintaoshao@tencent.com

**Keywords:** neural architecture search, evolutionary algorithm, individual interaction, training-free

## Abstract

Neural Architecture Search (NAS) has made significant advancements in autonomously constructing high-performance network architectures, capturing extensive attention. However, a key challenge of existing NAS approaches is the intensive performance evaluation, leading to significant time and computational resource consumption. In this paper, we propose an efficient Evolutionary Neural Architecture Search (ENAS) method to address this issue. Specifically, in order to accelerate the convergence speed of the algorithm and shorten the search time, thereby avoiding blind searching in the early stages of the algorithm, we drew on the principles of biometrics to redesign the interaction between individuals in the evolutionary algorithm. By making full use of the information carried by individuals, we promoted information exchange and optimization between individuals and their neighbors, thereby improving local search capabilities while maintaining global search capabilities. Furthermore, to accelerate the evaluation process and minimize computational resource consumption, a multi-metric training-free evaluator is introduced to assess network performance, bypassing the resource-intensive training phase, and the adopted multi-metric combination method further solves the ranking offset problem. To evaluate the performance of the proposed method, we conduct experiments on two widely adopted benchmarks, NAS-Bench-101 and NAS-Bench-201. Comparative analysis with state-of-the-art algorithms shows that our proposed method identifies network architectures with comparable or better performance while requiring significantly less time.

## 1. Introduction

The field of machine learning is undergoing an unprecedented boom [1], driven by pioneering networks such as Res-Net [2], VGG [3], and DenseNet [4]. These networks have played a pivotal role in yielding breakthrough results across a spectrum of application domains, encompassing image classification [5], medical image analysis [6], vegetation remote sensing [7]. But the success achieved by neural networks in these fields relies on the expert experience and domain-specific knowledge possessed by neural network designers, and this knowledge is not easily and quickly obtainable. The process of adjusting neural networks also leads to a significant waste of time and resources due to repeated experiments. This makes the manual design of neural networks challenging and inefficient [8].

To address the challenges arising from the manual design of neural networks, researchers have developed neural architecture search algorithms (NAS) [9], with the anticipation of autonomously obtaining excellent neural network architectures for the target domain, without the need for manual intervention. Neural architecture search primarily encompasses three aspects [10]: search space, search strategy, and performance evaluation. Search algorithms explore the search space to discover networks with superior performance, which is evaluated during the performance evaluation phase. Therefore, neural network architecture search can be modeled as an optimization problem aiming to achieve optimal performance. NAS has also found applications in various domains, such as medical image segmentation [11], video super resolution [12], facial expression recognition [13].

Based on different search strategies, neural network architecture search algorithms can be categorized into three types: reinforcement learning (RL)—based [14], gradient descent (GD)—based [15], and evolutionary computation(EC)—based [16]. In RL-based algorithms [17,18], the actions of the agent correspond to the generation of neural network architectures. The action space corresponds to the search space, and the reward corresponds to the accuracy of the designed architecture. Through the reward, RL can train the controller to prefer architectures with higher accuracy in the next iteration. The extensive evaluation process makes RL-based methods consume significant computational resources, resulting in high search costs. Despite recent efforts to improve through multi-agent parallelism [19], the performance remains unsatisfactory. In comparison to random search and reinforcement learning, GD-based methods exhibit better efficiency in search. This approach, through continuous relaxation, allows researchers to transform the discrete search space into a continuous space, enabling the use of gradient descent to search for architectures. Evidently, GD-based methods offer a substantial advantage in terms of computational speed. However, these methods are sensitive to initialization, implying the need for strong domain knowledge when constructing the initial search space. Additionally, due to their inherent alternating optimization characteristics, they may lead to performance collapse [20,21].

Compared to other methods, EC-based methods are less restricted by search space requirements, more automated, and insensitive to gradients, and have been widely adopted as search algorithms. Specifically, [22], as a pioneer of ENAS, adopts genetic algorithms as a search algorithm. The previous tournament selection operator was modified in [23] to discard older individuals in the population. Two levels of particle swarm optimization are used to evolve the whole network architecture in [24,25] proposes a new encoding method and crossover operator adapted to the need of variable length. In these methods, the main computational resource consumption is focused on the evaluation process of the network, and some improvements have been proposed in the subsequent methods, ref. [26] adopts an incremental evaluation to shorten the evaluation process, ref. [27] adopts the weight inheritance to reduce the cost of the evaluation, and [28] reduces the number of evaluations by the similarity evaluation of the individuals. However, in most of these works, there is an improvement of the operator without considering the relationship between individuals in the population and the utilization of the relevant information carried by the individuals in the problem. This means that in the early stages of the search, the search space is less utilized and time is wasted in the process of blind exploration.

Furthermore, when EC is employed as a search algorithm, it usually involves a large number of performance evaluation processes, which leads to high search costs that hinder the development of related research, among the algorithms mentioned above [22,23], consumed thousands of GPU Days (the number of GPUs used multiplied by the number of days searched) to get the results of the search. Subsequent methods have been proposed to try to solve this problem, e.g., weight inheritance [29]; early stopping [30]; low fidelity [31]; however, in these methods, training evaluation of the individuals is still required, and the acceleration results are not satisfactory, and usually the search process still takes several GPU days. Evaluation methods that do not require training have been applied to the evaluation process in NAS with the development of neural network interpretability. These methods [32,33] avoids the expensive training process by scoring the network at the initialization stage of the network, but in neural networks, the factors that affect the performance are more complex. Therefore the accuracy of the evaluation is not good. Meanwhile, in previous work, these methods only generate initial populations to reduce part of the evaluation process or only combine with simple evolutionary algorithms [34]. This means that the search process is slower to converge, less capable of global search, and consumes more time and computational resources. Meanwhile, in previous methods, the network is usually evaluated using only a single evaluation metric [32,35], which is not comprehensive enough to assess the network performance. Alternatively, multiple network metrics are used for assessment, but the rankings are directly added to combine the metrics [36], resulting in a situation where the rankings in multiple metrics conflict and the impact of the worse metrics is canceled out by the better metrics. Moreover, ref. [37] have reviewed the progress of zero-cost NAS and highlighted open challenges such as metric instability, search space bias, and poor generalization across datasets.

In order to solve the problems mentioned in the above content, we design a new efficient evolutionary neural network architecture search method without training for automatic neural network architecture search, which improves the search efficiency while ensuring the performance of the search results. Specifically, the method proposed in this paper is improved in terms of both algorithm and evaluation method. In the algorithm, the role of individuals in the population is emphasized, and the interactions among individuals in the population are enhanced, so that the relevant problem information carried by individuals can be further utilized and diffused through competition and cooperation among individuals to promote faster convergence of the algorithm. In the evaluation method, all the training and evaluation processes are omitted, and the performance of the network is evaluated at the initialization stage of the network. In order to improve the accuracy of the evaluation, the evaluation function is improved to assess the network performance more comprehensively from three aspects to reduce the possibility of misjudgment of the fitness function for the network performance, and a new way of combining the indicators is proposed to solve the problem of ranking discrepancy, which overall improves the consistency of the ranking between the fitness function and the actual performance.

The contributions of the proposed algorithm are summarised as follows.

- In order to improve the search efficiency of the algorithm, the proposed algorithm improves the interaction between the individuals, which drives the whole evolution in the direction of goodness and makes greater use of the problem information carried by each individual. This advancement facilitates faster convergence of the search algorithm.
- We propose a fitness function based on the combination of multiple zero-cost proxies that evaluates network architecture performance from multiple aspects and combines them. Compared with previous approaches, this paper considers more comprehensive performance influences, combines them in a more effective way, and evaluates them more accurately.
- Experiments on widely adopted benchmarks demonstrate that this paper’s method outperforms existing NAS methods and is able to find better performing network architectures in a shorter period of time. The effectiveness of this paper’s method is also demonstrated by ablation study.

The remainder of this paper is organized as follows. Section 2 provides an overview of the related work in the field of neural architecture search. In Section 3, we present the methodology employed in this research, providing a detailed description of the proposed method. Section 4 presents the experimental setup, results, and analysis, comparing them with previous studies and discussing their implications. Finally, Section 5 we will summarize the whole work and discuss the future directions for further research.

## 2. Related Works

In this section, we review related work in the field of neural architecture search, focusing on two key aspects: EC-based NAS methods and accelerated evaluation strategies.

### 2.1. EC-Based NAS

Due to their high flexibility, simplicity in concept, strong search capabilities, NAS methods based on Evolutionary Computation are widely embraced [38].

In EC-based NAS approach, a set of architectures is updated by continuous iteration to get the final network architecture. Typically, population initialization is performed to get the initial population, one or more parents are selected to generate new individuals through crossover and mutation operators, and well-performing individuals will be added to the population.

The variations among algorithms primarily stem from their approaches to constructing these evolutionary operations. LargeEvo [22] is often regarded as the pioneering EC-based NAS algorithm, employing genetic algorithms for automated search. In [23], aging evolution was introduced, Discarding older individuals during evolution forever keeps the population young and facilitates the exploration of more individuals. Ref. [39] Fusing the two operators while discarding the oldest and worst individuals. Furthermore, algorithms design their own crossover and mutation operators. Refs. [40,41,42] employ single-point crossovers [43], while [44] utilizes disruptive crossovers. Or have multiple crossover operators at the same time and randomly choose the type of crossover when an individual makes a crossover [45]. Mutations are usually performed at two levels [46], modifying the types of basic structures in the architecture and modifying the connectivity relationships. In [47,48], RNNs are trained to guide mutation operations. In addition to using GA as a search algorithm, recent studies have begun to use DE as a search algorithm because the DE algorithm has the advantages of fewer parameters and faster convergence. The standard DE algorithm is used as the search strategy in [49], which proves the superiority of DE applied in NAS. Ref. [50] combines the single-path coding in PSO [51] with DE, while adding the quadratic crossover [50] combines single-path coding in PSO [51] with DE, while adding secondary crossover. The coding strategy divided into two parts is designed in [52], and the local variation [53] applies DE to NAS by combining the individual selection strategy with the environment selection strategy in [54]. In all these works, DE has shown good performance, but the problem-related information carried by individuals in the population has not been further exploited, so it is necessary to further improve the DE algorithm to enhance the performance of the NAS algorithm.

### 2.2. Methods for Accelerated Evaluation

The development of neural network architecture search methods aims to obtain better performing networks in a shorter time, and a large amount of time in the architecture search process is consumed in the evaluation process [55], so it is particularly important to accelerate the evaluation process.

Early stopping strategy is used in [30], which is different from the previous full training, the training process in the early stopping strategy stops after a few epochs, and the training time is shortened by decreasing the number of epochs for training. Ref. [56] is accelerated by low fidelity estimation, which employs a small number of datasets to train to get the performance estimation. Ref. [57] avoided training of the network by performance predictor and predicted to get the performance of the network, but the performance predictor usually needs training process with data. Ref. [58] through network morphism the children inherit the weights of the parents, thus reducing the small part of training and achieving speedup. Through weight-sharing [20], a large supernet is obtained by training first, which makes the child network inherit weights from the supernet without training. However, all of these acceleration methods still leave the training process more or less intact, so there is still room for development for acceleration.

An ideal approach for accelerating evaluation is to obtain network performance without training, termed as zero-shot or training-free methods. In such methods, there is no need to train the network, and no trained agent is required for evaluation, making it extremely cost-effective. These approaches primarily evaluate networks from two aspects: parameters and structure. Parameter evaluation is mainly used for pruning, assessing the importance of parameters, such as fisher [59] and SynFlow [60]. Architecture-based methods, on the other hand, leverage the theory of Neural Tangents Kernel (NTK) to score networks based on attributes that enhance structural performance, as seen in NASWOT [32] and TE-NAS [33]. However, most zero-cost proxies do not accurately predict neural network performance.

In the process of combining with these metrics, different search strategies are used, for example, pruning is used as a search strategy in ZCNAS [61] and TE-NAS [33], Bayesian optimization is used in BANANA [62], evolutionary algorithms are used in NASWOT to warm-up subsequent searches, REA [23] is used as a search strategy in TEG-NAS [36], and TNASSE [63] uses the search economic algorithm as a search strategy, as stated before, in the process of combining with metrics that do not require training, most of the evolutionary algorithms used are simple evolutionary algorithms or are only used as a warm-up session, so the potential of combining evolutionary algorithms with metrics that do not require training is worth exploring.

## 3. Approach

The proposed method in this paper aims to search for neural network architectures with high accuracy within a sho-rter time consumption and lower computational consumption. This section explains the proposed method in detail. Specifically, we will begin by introducing the definitions of the environment and the local environment. Subsequently, we present the definition of individuals, exemplifying the encoding method for individuals within the NAS-Bench-201 search space. Following this, we introduce the overall algorithmic flow. Further, we delve into comprehensive explanations of the operations involved in the algorithm. Finally, we introduce the improved evaluation method proposed in this paper.

### 3.1. Problem Definition

The goal of this paper is to search for a high-performing neural network architecture a∈A from a predefined search space A, without requiring any network training during the search. Formally, this can be formulated as the following optimization problem:

(1) $$a^{*}=\arg\max_{a\in A}\;F\left(a\right)$$

where F(a) is the performance estimation function that evaluates architecture *a* without full training (e.g., using zero-cost proxies), and A is the discrete architecture search space defined by a specific benchmark, such as NAS-Bench-101 or NAS-Bench-201.

Our objective is to efficiently explore A to find the architecture $a^{*}$ with the highest predicted performance, by designing a novel evolutionary strategy incorporating both individual interactions and multi-source evaluation metrics.

### 3.2. Environment Definition

In order to enhance the interaction between individuals and further utilize the problem information carried by individuals, we adopt a different approach from traditional evolutionary algorithms. Specifically, we define a separate environment in which individuals interact with their neighbors.

**Figure 1:**
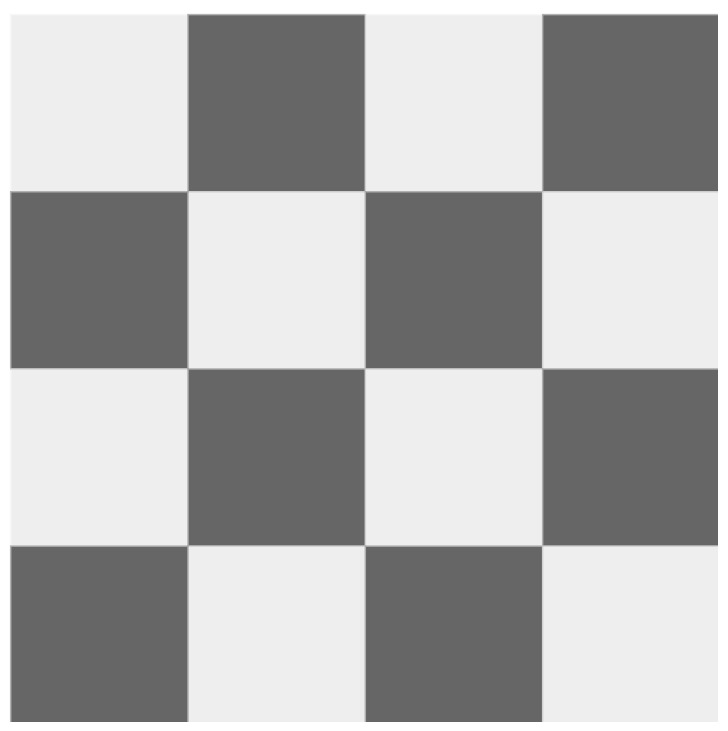
Diagram of environment for $L_{size}=4$.

The environment can be conceptualized as a grid-like structure resembling an alternating black and white chessboard (Figure 1). The size of the environment is determined by the integer $L_{size}$ ($L_{size}$ is specified as an even number), which corresponds to $L_{size}\times L_{size}$. Each individual is positioned at a fixed chessboard cell, and the size of the environment is equal to the number of chessboard cells, which is also equal to the number of individuals. As shown in Figure 1, if $L_{size}$ is 4, the environment size would be 4×4, and correspondingly, there would be 16 individuals located at 16 chessboard cells.

**Figure 2:**
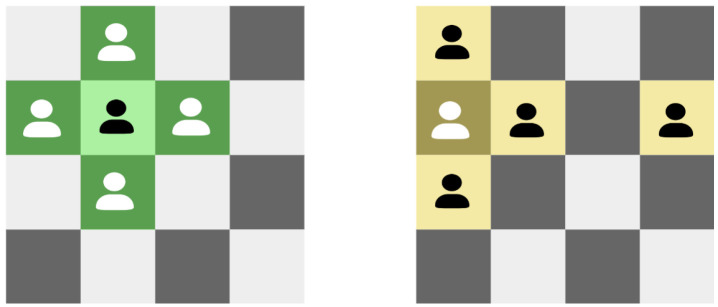
Diagram of local environment. (**left**): individual is not on the edge. (**right**): individual is on the edge.

Individuals located on chessboard cells only interact with their neighbors, and information is diffused through these interactions. In other words, each individual operates within its local environment, which consists of the individual itself and its four neighboring individuals of the environment. As shown in Figure 2, for non-edge individuals (depicted in light green), their neighbors include the four individuals located above, below, to the left, and to the right. When individuals are located at the edges (as depicted in dark yellow in the figure), it can be considered that each row and column forms a cyclic queue. Therefore, for an individual $A_{i,j}$, its neighbors $A_{i,j}^{neighbors}$ = { $A_{i^{\prime },j}$, $A_{i^{\prime \prime },j}$, $A_{i,j^{\prime }}$, $A_{i,j^{\prime \prime }}$ }.where

$$i’=\left\{\begin{array}{cc} i-1 & i\neq \;1\\ L_{size} & i=1 \end{array}\right.\;j^{\prime }=\left\{\begin{array}{cc} j-1 & j\neq \;1\\ L_{size} & j=1 \end{array}\right.i^{\prime \prime }=\left\{\begin{array}{cc} 1 & i=L_{size}\\ i+1 & i\neq L_{size} \end{array}\right.\;j^{\prime \prime }=\left\{\begin{array}{cc} 1 & j=L_{size}\\ j+1 & j\neq L_{size} \end{array}\right.$$

Since each individual can only perceive its local environment, competitive and cooperative behaviors occur solely between an individual and its neighbors. individuals interact with their neighbors to transmit information to them. In this way, information spreads throughout the entire environment. It can be observed that the model of local environment interaction is closer to the real evolutionary mechanisms found in nature than the population model in traditional genetic algorithms.

### 3.3. Individual Definition

In our approach, each individual represents a potential solution to the NAS problem. The quality of an individual is determined by its fitness function value, where higher values indicate better performance and a higher likelihood of survival in the environment. Thus, individuals strive to maximize their fitness function levels. We will delve into the fitness function details in upcoming sections.

When conducting a search, it is necessary to encode individuals to facilitate the subsequent search process. In NAS-related work, the search space is often divided into macro-skeleton and micro-cell levels. The macro-skeleton remains constant, focusing the encoding on the cell. As an example, we’ll discuss our encoding strategy using NAS-Bench-201 [64].

**Figure 3:**
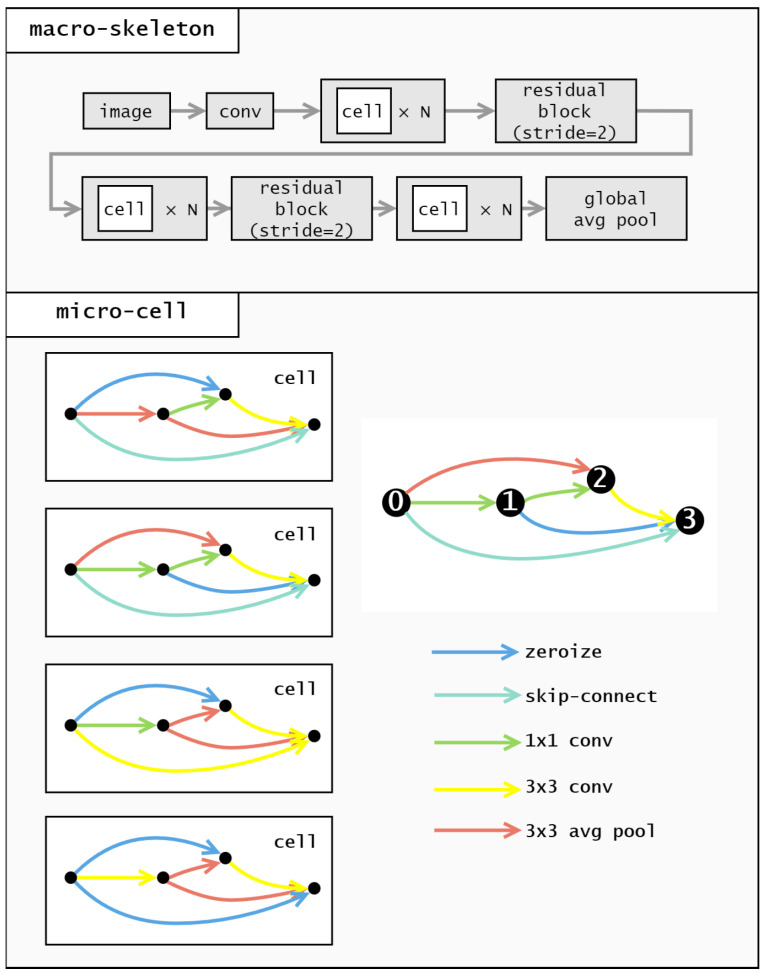
Architecture of NAS-Bench-201.

In NAS-Bench-201, the cell structure consists of four nodes, numbered 0, 1, 2, and 3, as shown in Figure 3. These nodes are connected in a unidirectional manner, resulting in six possible connections. This means the encoding length for a cell is 6.

**Figure 4:**
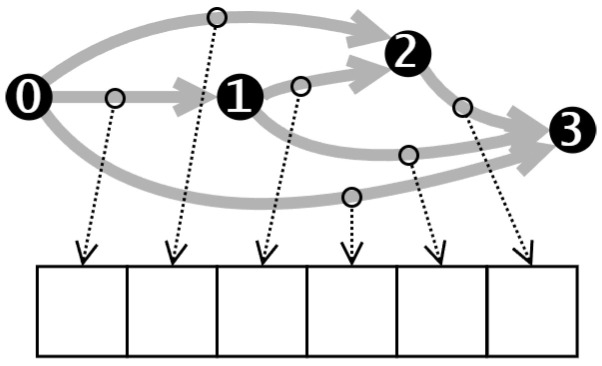
Encoding of NAS-Bench-201.

The cell encoding describes operations between different nodes. Each digit in the encoding corresponds to a specific node pair, as illustrated in Figure 4.

Therefore, the search space on NAS-Bench-201 can be expressed as:

(2) $$\begin{array}{c} A=\{A|A=\left(a_{1},\ldots ,a_{n}\right)\},\;n=6,\\ a_{i}\in \left[0,5\right],\;i=1,2,3,\ldots ,n \end{array}$$

**Figure 5:**
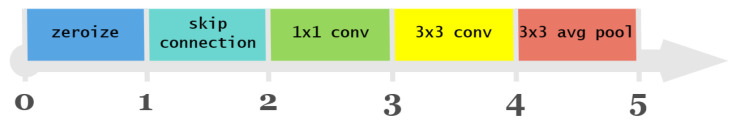
Mapping of real numbers to operators.

We employ a real number encoding method, as depicted in Figure 5, which involves mapping real number parameters back to operators during decoding. To maintain diversity and facilitate exploration, each digit in the encoding is a real number within the range of 0 to 5. Using real numbers instead of integers prevents the population from becoming too homogeneous, as integers would lead to numerous duplicates in the population and hinder the effectiveness of Differential Evolution (DE) in exploring the solution space.

(3) $$\mathrm{Map}\left(a_{i}\right)=\left\lfloor a_{i}\right\rfloor ,\;a_{i}\in \left[0,5\right]$$

The result of map indicates the type of operation between two nodes. Specifically, zeroize, skip-connect, 1 × 1 convolution, 3 × 3 convolution, and 3 × 3 average pooling are represented by the values 0, 1, 2, 3, and 4, respectively.

**Figure 6:**
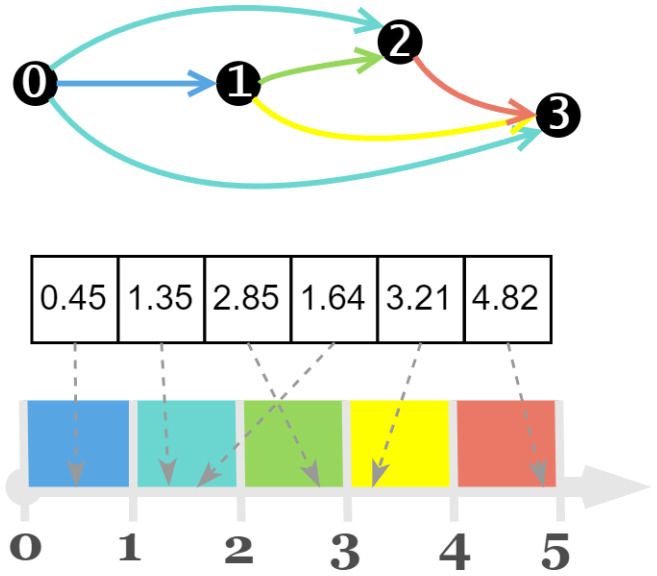
Example of encoding.

For example, let’s consider the cell shown in Figure 6. The corresponding encoding for this cell is [0.45, 1.35, 2.85, 1.64, 3.21, 4.82], and after the mapping operation of the Formula (3), the result obtained is [0, 1, 2, 1, 3, 4], indicating that the operations between the nodes are as follows: from the node 0 to node 1, a zeroize operation is applied; from the node 0 to node 2, a skip-connection is applied; from the node 1 to the node 2, a 1 × 1 convolution is applied; from node 0 to node 3, a zeroize operation is applied; from node 1 to the node 3, a 3 × 3 convolution is applied; and from node 2 to the node 3, an average pooling operation is applied.

This encoding strategy allows for a concise representation of the cell structure and the operations between nodes, facilitating the exploration and evaluation of different cell architectures in NAS-Bench-201.

### 3.4. Overall Introduction

In this section, the overall process of an efficient search algorithm proposed for evolutionary neural network architecture search is presented.

In the context of neural network architecture search, each individual represents a distinct network, and the population exists within the environment previously described. We represent the environment in which the individuals reside as *L*, where the size of the environment is determined by the integer $L_{size}$, corresponding to $L_{size}\times L_{size}$. This implies that the population size remains constant in each generation, with a fixed size of $L_{size}\times L_{size}$. After completing the search in each generation, a total of $L_{size}\times L_{size}$ neural networks are retained. We denote $L^{t}$ as the environment at the *t*-th generation, $Best^{t}$ is the best individual among $L^{0}$, $L^{1}$, $L^{2}$, …, $L^{t}$, and $CBest^{t}$ is the best individual in $L^{t}$.

Detailed explanations of the following operational operators will be provided in Section 3.5.

**Initialization**: The proposed method commences with the random initialization of the environment $L^{0}$. In this process, random sampling within the search space is utilized to select $L_{size}\times L_{size}$ neural networks, forming the initial population. Additionally, the individual with the highest fitness function value in the initial population is identified and designated as Best^0^. The method for computing fitness will be detailed in subsequent sections.

**Neighborhood Competition**: Subsequently, individuals in the environment interact with each other, facilitating information exchange. We apply the neighborhood competition operator for each individual in the environment $L^{t}$. This operator makes interaction between the individual and its local environment, leading to the exchange of information. Upon completion of the neighborhood competition, the current environment state is denoted as $L^{t+1/3}$.

**Global Exploration**: After completing the neighborhood competition, we employ the differential operator for global exploration. Within $L^{t+1/3}$, involving mutation and crossover operators. Perform the mutation operator to each individual $A_{i,j}$, yielding the mutated individual $A_{i,j}^{mutant}$. Subsequently, perform the crossover operator to generate the trial individual $A_{i,j}^{trial}$. If $\mathrm{Fitness}\left(A_{i,j}\right)<\mathrm{Fitness}\left(A_{i,j}^{trial}\right)$, replace $A_{i,j}$ with $A_{i,j}^{trial}$. By conducting global exploration, we can uncover superior neural network architectures, advancing the overall quality of the population. Once the process is finished for all individuals within $L^{t+1/3}$, it yields the environment $L^{t+2/3}$.

**Self-Learning**: After completing the differential global exploration, we perform self-learning on the best individual $CBest^{t+2/3}$ in the current population $L^{t+2/3}$. Self-learning allows the individual to autonomously explore the surrounding area for better individuals. Here, we employ a simple greedy algorithm for self-learning. After completing the self-learning, we obtain a new individual $CBest^{t+1}$, and the current environment state is recorded as $L^{t+1}$. In other words, at this point, we have completed one iteration of the population.

The evolution process continues until the termination condition is met. Ultimately, the best network architecture is decoded from the individual $Best^{t}$.

### 3.5. Evolutionary Operators

Neighborhood competition and neighborhood crossover operators facilitate competition and cooperation, respectively. Mutation and self-learning operators implement knowledge-based behaviors.

#### 3.5.1. Neighborhood Competition Operation

There exists a competitive relationship among the individuals. For an individual $A_{i,j}$, in its local environment, there are only five individuals: individual $A_{i,j}$, and its four neighbors $A_{i,j}^{neighbors}$, the individual with the highest fitness value is denoted as $M_{i,j}$.

(4) $$M_{i,j}=\arg\max\left\{\mathrm{Fitness}\left(A\right)|A\in A_{i,j}^{neighbors}\right\}$$

**Figure 7:**
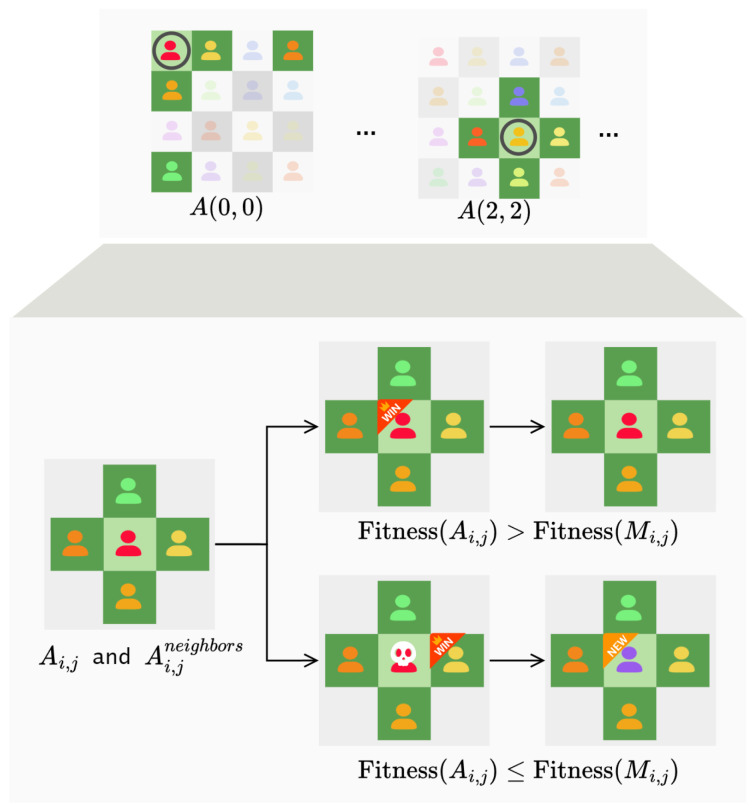
Neighborhoodcompetition operator.

There is a competitive relationship among all of them, like Figure 7 and an individual can survive in its local environment only when it has the maximum fitness value among them:

(5) $$\mathrm{Fitness}\left(A_{i,j}\right)>\mathrm{Fitness}\left(M_{i,j}\right)$$

If the individual $A_{i,j}$ satisfies (5), it can continue to survive in its current position. When an individual fails to satisfy (5), it will be removed from the environment. This process is similar to the selection operator in swarm intelligence algorithms. However, in swarm intelligence algorithms, the selection operator operates on the entire population, while the competitive operator occurs only among the individuals and their neighbors in this context.

In addition to the competitive relationships, there are also cooperative relationships among the individuals, which allow individual individuals to increase their fitness values. After the competitive operator is executed, if an individual fails to meet the fitness requirement, it will be removed from the environment, creating a corresponding vacancy. Individuals cooperate to generate new individuals, filling the vacant positions. New individuals can be generated through two different strategies. In the first strategy:

(6) $$A_{i,j}^{new}=Max_{i,j}+\mathrm{Rand}\left(0,1\right)\times \left(Max_{i,j}-A_{i,j}\right)$$

where $A_{i,j}^{new}$ represents the new individual generated to replace $A_{i,j}$, $Max_{i,j}$ represents the individual with the highest fitness value in the local environment $L_{i,j}$ where $A_{i,j}$ previously existed, and rand(0,1) denotes a random number drawn from the uniform distribution between 0 and 1.

The second strategy is as follows:

(7) $$A_{i,j}^{new}=Max_{i,j}+\frac{1}{t}\times G\left(0,1\right)$$

where *t* represents the current generation, and *G* denotes Gaussian noise. According to the assumption that “Good solutions are often found near good solutions”. In the operator, as the generation increases, the degree of mutation decreases, focusing the search near good solutions.

The two strategies for generating new individuals have different tendencies. Compared to the second strategy, the first one tends to emphasize cooperation between individuals. This is because we believe that among the killed individuals, there may also be factors beneficial to evolution. Thus, the first strategy combines the best individual with the current individual to generate a new individual, moving closer to the best individual in the local environment based on the current individual. The second strategy, on the other hand, leans more towards utilizing the best individual in the local environment to enhance the overall fitness value.

The overall neighborhood competition operator is shown in Algorithm 1.
**Algorithm 1** Neighborhood competition operations**Require:** $A_{i,j}\in R^{n}$: individual at position (i,j);$A_{i,j}^{\mathrm{neighbors}}\subset R^{n}$: neighbor individuals;$P_{s}\in \left[0,1\right]$: selection probability**Ensure:** $A_{i,j}^{\prime }\in R^{n}$: updated individual at position (i,j)    1:$M_{i,j}$← None    2:**for each** *A* **in** $A_{i,j}^{neighbors}$ **do**    3:    **if** Fitness(A) > $\mathrm{Fitness}(M_{i,j})$ **then**    4:        $M_{i,j}$←*A*    5:    **end if**    6:**end for**    7:**if** $\mathrm{Fitness}\left(A_{i,j}\right)<\mathrm{Fitness}\left(M_{i,j}\right)$ **then**    8:    **if** Rand(0,1) < $P_{s}$ **then**    9:         NewIndividual← **Strategy1( )**  10:                                                         ▹ Use Strategy 1 to generate new individual  11:    **else**  12:         NewIndividual← **Strategy2( )**  13:                                                         ▹ Use Strategy 2 to generate new individual  14:    **end if**  15:**else**  16:    NewIndividual$\leftarrow A_{i,j}$                                ▹ Retain the original individual  17:**end if**  18:**Return** NewIndividual as the updated individual $A_{i,j}$

#### 3.5.2. Global Exploration

New offspring are brought into existence via the implementation of mutation operations for each individual, employing a specific mutation strategy. In our works, we traverse through every individual denoted as $A_{i,j}$ residing on the grid. When a randomly generated number U(0,1) falls below a predefined threshold Pm, we proceed to initiate a mutation operation on that specific individual. The mutation operation is performed by selecting any two individuals from the environment, excluding the individual $A_{i,j}$, identified as $A_{k,l}$ and $A_{m.n}$, (i,j)≠(k,l)≠(m,n). These selected neighbors and individual $A_{i,j}$ itself, collectively form the three parents essential for executing the differential mutation operation. This process is used to generate a new individual as follows:

(8) $$A_{i,j}^{mutant}=A_{i,j}+F\cdot \left(A_{k,l}-A_{m,n}\right)$$

where $A_{i,j}^{mutant}$ is the mutant individual generated for individual $A_{i,j}$ in the environment, *F* is the scaling factor (which usually takes values within the range [0, 1]).

After the mutation, a crossover operation is applied to each individual $A_{i,j}$ and its corresponding mutant individual $A_{i,j}^{mutant}$ to generate a trial individual $A_{i,j}^{trial}$. We use a simple binomial crossover, which chooses the value for each dimension *i* from $A_{i,j}$ with probability Pc and from $A_{i,j}^{mutant}$ otherwise.

(9) $$a_{i,j}^{trial}=\left\{\begin{array}{cc} \; & a_{i,j},\;\mathrm{rand}\left(0,1\right)\leq Pc\\ \; & a_{i,j}^{mutant},\;otherwise. \end{array}\right.$$

#### 3.5.3. Self-Learning Operation

In addition to interacting with the environment, an individual can self-learn to improve its fitness value. According to [65], in the algorithm, individuals can improve the quality of their offspring through local refinement to achieve lifelong learning. Therefore, in our algorithm, we employ the corresponding local search operator to achieve individual self-learning.

In self-learning, the goal is to improve the ability of future generations by obtaining a better solution in the current environment than the current one, without necessarily reaching the current global optimum. Therefore, in this case, we use a simple greedy algorithm for self-learning.

The overall algorithm flow, as depicted in Algorithm 2, unfolds as follows. After executing the preceding operators, the algorithm identifies the best solution within the current population, which becomes the input for the local search as the current individual. The algorithm then applies the appropriate mutation operator to the current individual, generating the corresponding neighbor individual. Within the neighbor individuals, it seeks out the individual with the highest fitness value. If this individual’s fitness value surpasses that of the current individual, the current individual is replaced with the one with the highest fitness value. This process shares similarities with the competition operator discussed earlier, with the distinction lying in how the retained individuals are generated. Additionally, the mutation operator employed here differs from the one used earlier.

**Figure 8:**
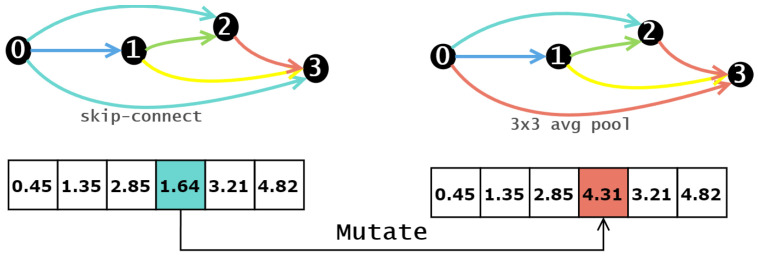
Mutate of self-learning operation.

In this context, the mutation operator is applied to neural network architecture design. Specifically, it involves a single-point mutation on the neural network architecture. Compared to other operators, single point mutation retains a higher degree of information about the current individual, which is in line with our aim of using self-learning operators. The mutation operator randomly selects a position within the encoding and replaces it with a random number from the range [0, 5]. This implies that within the network architecture, one operation will be replaced. As depicted in the Figure 8, an original skip connection in the network architecture is replaced with a 1 × 1 average pooling operation.
**Algorithm 2** Self-learning operation**Require:** $CBest^{t+2/3}\in R^{n}$: initial solution**Ensure:** $CBest^{t+1}\in R^{n}$: result solution    1:CurrentSolution ← $CBest^{t+2/3}$    2:CurrentFitness ← Fitness(CurrentSolution)    3:**while** stop condition not meet **do**    4:    Neighbors ← GenerateNeighbors(CurrentSolution)    5:    BestFitness ← CurrentFitness    6:    **for** Neighbor in Neighbors **do**    7:        NeighborFitness ← Fitness(Neighbor)    8:        **if** NeighborFitness > BestFitness **then**    9:             BestNeighbor ← Neighbor  10:           BestFitness ← NeighborFitness  11:        **end if**  12:    **end for**  13:    **if** BestFitness > CurrentFitness **then**  14:        CurrentSolution ← BestNeighbor  15:        CurrentFitness ← BestFitness  16:    **else**  17:        break  18:    **end if**  19:**end while**  20:$CBest^{t+1}$ ← CurrentSolution  21:**Return** $CBest^{t+1}$

### 3.6. Fitness Function

In evolutionary algorithms, the evaluation of individuals within a population relies on fitness functions, which indicate an individual’s adaptability to a specific environment, reflecting the quality of the represented solution. Correspondingly, each optimization problem may employ a distinct fitness function. In the context of NAS, classification accuracy is conventionally employed as the fitness measure for individuals. However, obtaining classification accuracy for a network typically entails costly network training. The aim of this paper is to discover high-performing neural network architectures without the need for network training during the search process. To address this challenge, we introduce a novel fitness function that eliminates the requirement for training during the search process, thereby better aligning with the aforementioned goals.

According to [33,66], NTK can be used to measure the effectiveness of optimizing a network using gradient descent. however the constant is computationally expensive and time consuming. According to [67,68], the constant NTK can be approximated on the mini-batch by the trace paradigm and the gradient flow, which greatly saves computational time. Therefore, when measuring the trainability of the network, we use the approximated NTK computed on mini-batch to get the performance of the network, being written as:

(10) $$S_{T}=\frac{d}{l}{∥\frac{1}{b}\sum_{j=1}^{b}Grad_{A}L_{x_{j}}∥}_{2}^{2}$$

where *d* is the size of the dataset indicating the total number of samples, *l* is the Lipschitz constant of the loss function used for normalizing the gradient’s contribution, *b* is the mini-batch size representing the number of samples used per iteration, ${\mathrm{Grad}}_{A}L_{x_{j}}$ is the gradient of the loss function *L* with respect to the architecture *A* for the *j*-th data point $x_{j}$, and ${∥\cdot ∥}_{2}$ denotes the $L_{2}$ norm representing the Euclidean length of the gradient.

It can be seen that the trace norm of NTK is related to the Frobenius norm of the Jacobian matrix [33], so a higher $S_{T}$ may have more parameters, which means better fitting ability, but the generalization ability may be poor. In contrast, we expect to obtain a neural network that is easy to converge while also having better generalization. Therefore, we expect a metric that can measure the network’s generalizability, that is, the performance that remains good when the network encounters unseen data, and a plain idea is that my network’s generalization is good when it thinks that two different inputs correspond to the same output. Therefore when evaluating the generalization performance of a neural network, we propose the following method:

First, for a given input image *x*, we generate a new image $x_{\mathrm{noisy}}$ by adding Gaussian noise. Inputting these two images separately into the neural network, we focus on and compare their feature maps output at the last pooling layer. Let the outputs of the last pooling layer of the network be F(x) and $F(x_{\mathrm{noisy}})$, respectively, and these feature maps can be regarded as the mapping of the function *F* on the input.

To quantify the differences between the feature maps, we use the Euclidean distance to define the distance between them:

(11) $$S_{G}=\sqrt{\sum_{i,j}{\left[F\left(x\right)\left(i,j\right)-F\left(x_{\mathrm{noisy}}\right)\left(i,j\right)\right]}^{2}}$$

This distance metric can help us to evaluate the difference in the response of the neural network to the addition of Gaussian noise to the input image, thus providing a quantitative analysis of the network’s generalization ability, especially in an untrained initial state.

The last factor in the fitness function is used to describe the network’s expressiveness, which refers to the network’s ability to approximate complex functions [69]. Higher network expressiveness leads to better overall performance. In this paper, we utilize the $S_{E}$ [70] to assess expressiveness. The $S_{E}$ consists of two components. The first part characterizes the network’s expressiveness using Gaussian complexity:

(12) $$\phi \left(x\right)=log\left(E_{x,\epsilon }{∥f(x)-f(x+\alpha \epsilon )∥}_{F}\right)$$

where x,ϵ∼N(0,1), ${∥•∥}_{F}$ stands for Frobenius norm, α is an adjustable hyper parameter, e.g., 0.01.

The second part is used for scale-sensitive problems, and the scale-insensitive effect is achieved by scaling the first part by the product of the variance statistics of the BatchNorm layer, So the complete $S_{E}$ is:

(13) $$S_{E}=\phi \left(x\right)+\sum_{i}log(\sqrt{\frac{\sum_{j}\sigma_{ij}^{2}}{m}}$$

$\sigma_{ij}$ is the standard deviation of the *i*th channel in *i*th BN layer with *m* output channels.

The rationale for selecting these three specific components is to create a holistic evaluation metric. $S_{T}$ serves as a proxy for the network’s trainability, indicating how readily the architecture can be optimized. $S_{G}$ is designed to estimate generalization by measuring the network’s robustness to input perturbations, a key quality for performance on unseen data. Finally, $S_{E}$ quantifies the network’s expressiveness, or its capacity to model complex functions. By synergizing these three complementary aspects, our fitness function aims to provide a more accurate and robust ranking of architectures compared to relying on any single zero-cost proxy.

The last question is how to combine the three indicators. In multi-criteria evaluations, we encountered the issue where each criterion $S_{T}$, $S_{G}$, and $S_{E}$ correlates differently with the ground truth, causing significant discrepancies in a model’s rankings across different metrics. Simple normalization and summation, or independently ranking and summing each criterion, might lead to extreme rankings offsetting each other, failing to accurately reflect the model’s overall performance. To address this, we chose to process the rankings of the criteria using logarithmic and exponential transformations. This method mitigates the impact of extreme rankings by first applying a logarithmic transformation to the rankings, followed by an exponential reversal, resulting in a more stable and balanced aggregate score.

(14) $$S\left(i\right)=\sum_{M\in \{T,G,E\}}e^{-\log(\mathrm{Rank}\left(S_{M}\left(i\right)\right)+\epsilon )}$$

where ϵ is a small positive constant to avoid logarithms of zero. This approach effectively provides a balanced composite ranking, accommodating the varying influences of each criterion on the overall assessment.

## 4. Experiments and Results

To validate the effectiveness of the proposed algorithm, experiments were conducted on two widely adopted benchmarks: NAS-Bench-101 [71] and NAS-Bench-201 [64]. In this section, we will first introduce these two benchmarks, the corresponding datasets, and peer competitors in the field. Then, we will describe the relevant parameters used in the experiments and finally present the experimental results and analysis.

### 4.1. Benchmark Datasets

NAS-Bench-101 and NAS-Bench-201 are two widely used benchmarks in the field of neural network architecture search. These benchmarks provide researchers with standardized and pre-evaluated architectures to facilitate the comparison and evaluation of different search algorithms.

In both NAS-Bench-101 and NAS-Bench-201, the search space is constructed based on cells, which are fundamental building blocks of neural networks. These cells are formed by stacking repeated modules, resembling the structure of well-designed manually crafted networks. This fixed search space allows researchers to explore and evaluate a diverse range of architectures.

In NAS-Bench-101, the search space consists of 3 stacked cells, forming a neural network. Each cell is composed of 7 nodes, where the first node is fixed as the input node and the last node is fixed as the output node. The maximum number of edges in a cell is limited to 9. Each node in the cell represents an operation, and the available operations include 3 × 3 convolution, 1 × 1 convolution, and 3 × 3 max pooling. The benchmark provides a collection of 423,624 candidate neural networks, and these architectures are trained and evaluated on the CIFAR10 dataset.

On the other hand, NAS-Bench-201 defines a search space with 4 nodes and 6 edges. The edges represent the possible operations between two nodes, and the available options include zeroize, skip-connect, 1 × 1 convolution, 3 × 3 convolution, and 3 × 3 average pooling. With the fixed size of the cell and the predetermined set of operations, there are a total of $5^{6}=15625$ candidate cell architectures. The benchmark provides training data and performance evaluations on CIFAR10, CIFAR100, and imagenet16-120 datasets for all these candidate architectures.

While both benchmarks serve the purpose of evaluating neural network architectures, there are some differences between NAS-Bench-101 and NAS-Bench-201. NAS-Bench-101 focuses on training architectures on a single dataset (CIFAR10), but it offers a larger search space compared to NAS-Bench-201. On the other hand, NAS-Bench-201 provides training data and performance evaluations on multiple datasets, allowing researchers to assess the generalization capability of architectures.

### 4.2. Peer Competitors

To validate the effectiveness of the proposed method, we conducted comparisons with other state-of-the-art techniques in our experiments. As discussed in Section 2, the methods chosen for comparison should cover RL, EA, and gradient-based approaches. Within these categories, most gradient-based methods have achieved acceleration through weight sharing, which is a prominent research direction in NAS. Consequently, the methods selected for comparison are categorized into “weight sharing” and “non-weight sharing”. Additionally, as we employ a training-free evaluation approach in this paper, we also compared our results with training-free methods. Furthermore, we included manually designed neural networks and random search as baseline approaches in the comparison.

Specifically, the first category of non-weight sharing work includes manually designed neural networks (ResNet) [2], random search with parameter sharing (RSPS) [72], regularized evolution for architecture search (REA) [23], and Bayesian optimization with hyperband (BOHB) [73], Assisted Regularized Evolution (AREA) [32].This basically covers all non-weight-sharing search methods mentioned in the related works.

The second category of methods is known as weight-sharing based methods. These methods include various techniques such as differentiable architecture search (DARTs-v1, DARTs-v2) [15], subgraph-based differentiable architecture search (GDAS) [74], neural architecture search via self-evaluated template network (SETN) [75], efficient neural architecture search (ENAS) [76], beta-decay regularization for differentiable architecture search [77] (β-DARTS). Among these methods, DARTs serves as the baseline for all the mentioned works.

The third category comprises training-free methods that avoid the computational costs associated with network training, allowing a more focused search process. This category includes methods such as Guided Evolutionary Neural Architecture Search (GEA) [78], Neural Architecture Search Without Training (NASWOT) [32], Training-free NAS (TE-NAS) [33], TEG-NAS [36], TNASSE [63], Green Neural Architecture Search (KNAS) [79], and Zero-Cost Proxies for NAS (ZCNAS) [61]. Among them, TE-NAS and ZCNAS use pruning as a search strategy, NASWOT, KNAS use random search as a search strategy. TNASSE, GEA, TEG-NAS, use evolutionary algorithms as a search strategy.

### 4.3. Parameter Setting

The experiments are implemented using Python 3.8 and PyTorch 1.7.0 on a PC with a single Nvidia RTX 3060 GPU. The hyperparameters (such as learning rate, batch size, etc.) are set according to the configurations provided in [64,71], to eliminate the influence of hyperparameters in the comparative experiment results. The parameters used in the algorithm are kept mostly consistent with the reference [49,80], population size set of 16, and 20 generations. To avoid the algorithm becoming a random search, the differential rate and crossover rate are set to 0.6 and 0.45, designed to foster a healthy balance between exploring new regions of the search space (exploration) and refining existing solutions (exploitation). A higher differential rate encourages the generation of diverse candidate solutions, while the crossover rate ensures that beneficial architectural motifs are preserved and propagated. In the self-learning operator, the Gaussian noise was configured with a mean of 0 and a standard deviation of 1. This standard normal distribution is a typical choice for introducing small, unbiased random perturbations, enabling fine-grained local search around promising architectures. Following the common conventions in evolutionary algorithms. In the self-learning operator, the number of neighbors is 8 and the number of iterations is 5. To avoid any errors in the code execution, the results of the peer competitors in the experiments are taken directly from the relevant published papers.

### 4.4. Results and Analysis

#### 4.4.1. Overall Results

Because the goal is to be able to search for better performing neural network architectures faster, the focus of the comparison will be on the algorithm’s search time and the network performance obtained from the search (in the case of classification tasks, this is represented as classification accuracy). The search time will be expressed in terms of GPU days/hours/seconds, calculated by multiplying the number of GPUs used for the search by the search time. For example, if the search takes 3 days on two GPUs, the GPU days would be 6; if the search takes 60 s on two GPUs, the GPU seconds would be 120. The actual running time of the algorithm determines whether it is measured in GPU days or GPU seconds, but it should be consistent for comparison purposes.

We conducted four experiments with different random seeds to obtain experimental results and report the final averages and standard deviations. Similar to previous work, our approach for architecture search was performed on CIFAR-10. We obtained the final network encodings, which were then used to evaluate the performance on relevant datasets. In NAS-Bench-201, the relevant datasets included CIFAR-10, CIFAR-100, and ImageNet-16-120. In the table, we also present the test accuracy and validation accuracy for each dataset. Validation accuracy is obtained by training the model on the training dataset and evaluating it on the validation dataset. Test accuracy, on the other hand, is obtained after completing both the training and validation phases, through evaluation on a separate test dataset. Typically, these two accuracy metrics may not align.

Table 1 shows the results of the proposed algorithm in this paper compared with other well-performing competitors on NAS-Bench-201. From top to bottom, the first column consists of five non-weight sharing algorithms. Among them, ResNet is designed manually, while the other three are obtained through neural architecture search algorithms. The difference lies in the search strategies used, namely random search, Bayesian optimization, and evolutionary algorithm. The second column consists of six weight sharing algorithms, where most of them use gradient-based search strategies. The third column consists of seven training-free algorithms, where the difference lies in the zero-shot proxies used and the search strategies employed. Most of the competitors’ metrics are directly taken from the NAS-Bench-201 paper.

Validation accuracy and test accuracy are both provided in the table, but in the subsequent sections, the term “accuracy” as mentioned will follow the convention in related work, referring to test accuracy. Search accuracy and search cost are the metrics of our primary interest.

**Figure 9:**
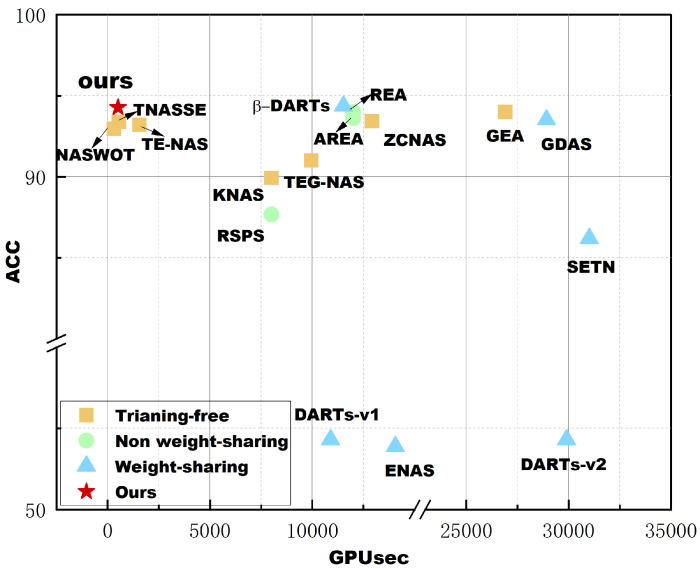
Test accuracy VS search cost in NAS-Bench-201.

The precision of validation and testing are provided in the table, but in the following text, we will refer to the accuracy mentioned in related work, which refers to test accuracy. Search precision and search cost are the indicators we care about. We created a scatter plot as Figure 9 using the test accuracy and search cost data from Table 1 on CIFAR-10 to facilitate a clearer comparison of the algorithms. From the plot, we can see that in terms of search cost, our method outperforms all non weight sharing and weight-sharing algorithms, and is better than all training-free algorithms except NASWOT. However, compared with NASWOT, our algorithm achieves better network accuracy, and the standard deviation of the results is smaller, indicating that the algorithm is more stable. In terms of accuracy, the algorithm proposed in this paper also shows excellent performance, only lagging behind DARTs, but the time we need is considerably shorter compared to DARTs. Furthermore, an interesting finding is that among the weight sharing methods compared, almost all of them, except DARTs, have poor accuracy. This may be due to the presence of too many jump connections in the common architecture of these algorithms, while the structure on NAS-Bench-201 was not specifically designed to fulfill this condition. As a result, many incorrect evaluations occurred, leading to performance crashes. We conducted another experiment on NAS-Bench-101 to validate the effectiveness of the proposed method on a larger dataset and compared it with two non-weight sharing, two weight sharing, and three training-free algorithms. The results are shown in Table 2. In terms of accuracy, ENAS achieved the best result, but at the same time, it consumed the most time among these algorithms. All training-free algorithms have an advantage in search cost. Meanwhile, among all training-free algorithms, our algorithm achieved the best accuracy.Another interesting finding is that the standard deviation of the training-free method is larger than that of the method requiring training, both on NAS-bench-201 and NAS-bench-101. We speculate that this is probably due to the fact that there is always a gap between the evaluation results given by the zero-cost proxy used in the training-free method and the true accuracy.

To quantitatively validate the performance differences observed in Table 1, we conducted an extensive experimental evaluation with 10 independent runs (N = 10) for our algorithm and key baseline methods. To statistically compare the performance, we employed the Wilcoxon signed-rank test, a powerful non-parametric method ideal for the typical data distributions found in NAS experiments. The analysis was performed on the paired test accuracy results from the CIFAR-10 dataset, using a two-tailed test with a standard significance level of α = 0.05. The results of our statistical analysis are summarized in Table 3.

The analysis revealed several key findings. First, the proposed method showed statistically significant and substantial improvements over the NASWOT and TEG-NAS baseline methods. Since the p-value was well below the 0.002 threshold and accompanied by a large effect size (r > 0.7), this proves that the proposed method is not a random coincidence.

Secondly, comparison with REA shows that there is no statistically significant difference between the two (*p* > 0.3). This result indicates that our method is statistically comparable to the current leading method in terms of final model accuracy. The moderate effect size indicates that, despite some minor differences, the overall performance capabilities are comparable. However, in terms of time cost, the method proposed in this paper is far superior.

#### 4.4.2. Abalation Study

In this section, we will delve into the details of each component of the proposed algorithm through ablation experiments, aiming to gain a deeper understanding of their specific impact on the final results. First we further validate the effectiveness of the proposed method on two benchmarks, NAS-Bench-201 and NAS-Bench-101, and then conduct experiments on NAS-Bench-201 to prove the validity of the components in the adopted evaluation approach.

First we compare the method proposed in this paper with the method that retains only the global search operator on two benchmarks. The specific results are displayed in the last two rows of Table 1 and Table 2, and ours- denotes the result of keeping only the global search operator. From the results, it can be seen that the search speed is faster when only the global search operator is retained, and also the results of the search are comparable with the other algorithms in the table, which indicates that the evaluation method we used and its combination are effective. Compared with the method proposed in this paper, the average accuracy has decreased, while the standard deviation has increased but not significantly, which may be due to the smaller dimension of NAS-bench-201, the same experiment is conducted on NAS-bench-101 with a larger dimension, and it can be seen that there is a significant difference in the standard deviation, and at the same time the average accuracy is lower than that of the original algorithm, and it is reasonable to judge that our proposed algorithm is more stable while improving the final result of the algorithm.

The next thing we want to further verify is that there is about the validity of the parts of the fitness function with the combination method. First we use the algorithm proposed in this paper, but replace the fitness function with $S_{T}$, $S_{G}$ and $S_{E}$ respectively to conduct experiments on the CIFAR10 dataset of NAS-Bench-201, and the experimental results are shown in Table 4.

Table 4 shows the specific results of the ablation experiments. As can be seen from the table, the best results, both in terms of mean precision and standard deviation, were achieved using a combination of three metrics compared to one metric alone. However, there is also some competition in the results obtained when one metric is used alone, which shows that there is some correlation between all three metrics involved and the groundtruth, as well as proving the effectiveness of the algorithm used.

**Figure 10:**
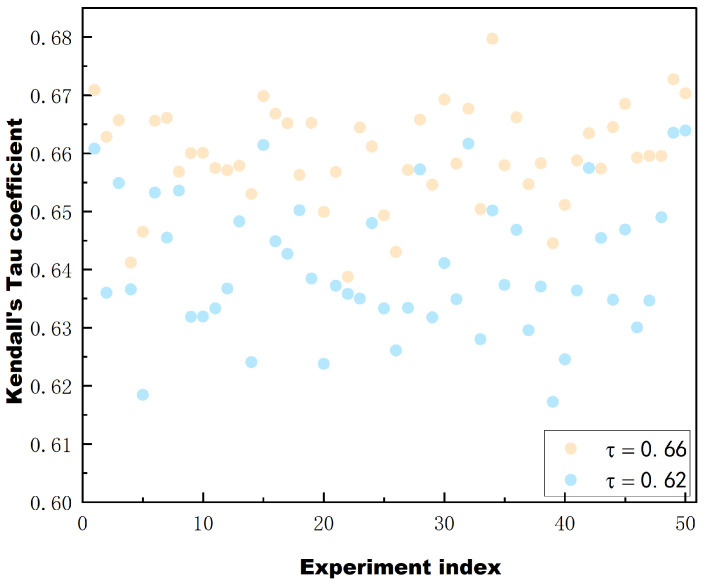
Kendall coefficients corresponding to 200 randomly sampled architectures with 50 experiments on NAS-bench-201, with the methodology of this paper in yellow and the rankings directly summed in blue.

To further validate the validity and reliability of the proposed fitness function as well as the combination approach, we randomly selected 200 network architectures from NAS-Bench-201. To prevent the results from being influenced by chance, we conducted 50 rounds of random sampling. Specifically, we used Kendall’s Tau correlation coefficients to measure the rank correlation between predicted and ground truth values.Kendall’s Tau compares the order of each pair of data points between the two variables and checks that their orders agree. If the order of the two variables is consistent, it indicates a high degree of correlation. Conversely, if the order is different, it indicates a lower correlation. In this study, higher Kendall’s Tau values indicate a higher degree of similarity between the rankings based on the fitness function and the actual classification accuracy rankings. Meanwhile, we compare the combination using the combination proposed in this paper and the combination by adding the ranks directly, and give the correlation Kendall’s Tau in Figure 10.

The results are shown in Figure 10. The horizontal axis represents the number of experiments, 50 in total, and the vertical axis represents the Kendall’s Tau correlation coefficients.The blue data corresponds to the combination of the rank values directly added together, while the yellow data corresponds to the combination proposed in this paper. As can be seen from the figure, Kendall’s Tau remains relatively stable in all 50 experiments, and the Kendall’s correlation coefficient corresponding to the combination method adopted in this paper is 0.66. This noteworthy consistency indicates the robustness and reliability of the value function. Furthermore, in the proposed algorithm, the agent’s evolution towards a better solution partially depends on the current optimal solution. This means that the agent considers the current optimal solution when trying to improve itself. By doing so, the agent can utilize the knowledge and insight gained from the current optimal solution to guide its search for better solutions. Therefore, we also tested the top 200 networks in terms of accuracy in the benchmark and the results show that Kendall’s Tau has a correlation coefficient of 0.68. This indicates that the value function used is reliable. The Kendall’s coefficient is higher for the combination approach used in this paper compared to the combination approach of adding the ranks directly, which further proves the validity of the combination approach used in this paper.

To further understand the dynamic behavior of the proposed algorithm, we analyze the convergence trend during the search process on the NAS-Bench-201 benchmark using the CIFAR-10 dataset.

**Figure 11:**
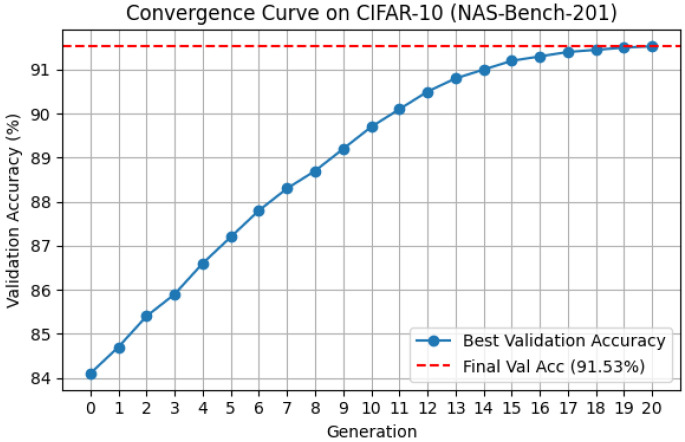
Convergence cure on CIFAR-10.

As illustrated in Figure 11, the validation accuracy of the best-found architecture improves steadily as the number of generations increases. Specifically, the initial population at generation 0 achieves an average validation accuracy of approximately 84.1%. In the early stages, the performance improves rapidly, reaching around 89.3% by generation 10. This rapid ascent is attributed to the neighborhood competition mechanism, which effectively guides the search towards promising regions of the search space by leveraging local peer interactions.

In the later stages, the improvement becomes more gradual, with the final best architecture achieving a validation accuracy of 91.53% and a test accuracy of 94.49%. This stable convergence is facilitated by the self-learning mechanism, which enables local refinement around the current best architecture using stochastic neighbor-based exploration.

Overall, the convergence curve exhibits a typical evolutionary optimization pattern: an initial rapid gain followed by diminishing improvements as the search approaches a local optimum. The smooth and monotonic improvement observed validates the effectiveness and stability of the proposed hybrid strategy combining both global exploration and local exploitation.

## 5. Conclusions

In this research, we introduce an efficient search algorithm for evolutionary neural network architectures while employing a score function as a zero-cost proxy for network evaluation. This not only enhanced the algorithm’s search capabilities but also reduced the evaluation process overhead, thus improving search efficiency. Experimental results indicate that our proposed method significantly reduces time consumption compared to other algorithms, while maintaining search performance, thanks to the designed algorithm and the employed score function. In future work, we aim to further enhance the algorithm by improving the encoding methods to better integrate with local search and enhance the accuracy and applicability of the score function in various scenarios.

While our experiments on NAS-Bench-101 and NAS-Bench-201 provide a controlled and reproducible validation, it is worth briefly discussing the broader applicability of our method. The proposed framework is designed for general-purpose use. Its core components—the evolutionary algorithm and the training-free fitness function—can be directly applied to a new, real-world task by simply defining a domain-specific search space and providing a small sample of the target data. This makes our approach particularly valuable for industrial or scientific applications with massive datasets, where the cost of traditional training-based NAS is a major barrier. Our method effectively democratizes NAS by making it computationally affordable and highly efficient.

## Figures and Tables

**Table 1:** Experimental results compared on NAS-Bench-201.

Algorithm	CIFAR10		CIFAR100		ImageNet16		Cost	Type
	**Valid**	**Test**	**Valid**	**Test**	**Valid**	**Test**	**GPUsec**	
ResNet	90.83	93.97	70.42	70.86	44.53	43.63	-	manual
RSPS	84.16 ± 1.69	87.66 ± 1.69	59.00 ± 4.60	58.33 ± 4.34	31.56 ± 3.28	31.14 ± 3.88	8007	Random search
BOHB	90.82 ± 0.53	93.61 ± 0.52	70.74 ± 1.29	70.85 ± 1.28	44.26 ± 1.36	44.42 ± 1.49	12,000	BO
REA	91.22 ± 0.25	93.97 ± 0.31	72.36 ± 1.07	72.14 ± 0.86	45.09 ± 0.92	45.55 ± 1.02	12,000	EA
AREA	91.20 ± 0.27	93.95 ± 0.39	71.95 ± 0.99	71.92 ± 1.29	45.70 ± 1.05	45.40 ± 1.14	12,000	EA
ENAS	37.51 ± 3.19	53.89 ± 0.58	13.37 ± 2.35	13.96 ± 2.33	15.06 ± 1.95	14.84 ± 2.10	14,059	weight sharing
DARTs-v1	39.77 ± 0.00	54.30 ± 0.00	15.03 ± 0.00	15.61 ± 0.00	16.43 ± 0.00	16.32 ± 0.00	10,890	weight sharing
DARTs-v2	39.77 ± 0.00	54.30 ± 0.00	15.03 ± 0.00	15.61 ± 0.00	16.43 ± 0.00	16.32 ± 0.00	29,901	weight sharing
GDAS	90.00 ± 0.21	93.51 ± 0.13	71.14 ± 0.27	70.61 ± 0.26	41.70 ± 1.26	41.84 ± 0.90	28,926	weight sharing
SETN	82.25 ± 5.17	86.19 ± 4.63	56.86 ± 7.59	56.87 ± 7.77	32.54 ± 3.63	31.9 ± 4.07	31,010	weight sharing
β-DARTS	91.55 ± 0.00	94.36 ± 0.00	73.49 ± 0.00	73.51 ± 0.00	46.37 ± 0.00	46.34 ± 0.00	11,520	weight sharing
GEA	91.26 ± 0.20	93.99 ± 0.23	72.62 ± 0.77	72.36 ± 0.66	45.97 ± 0.72	46.04 ± 0.67	26,911	training-free
KNAS	89.63 ± 1.78	89.92 ± 1.89	62.60 ± 3.69	62.73 ± 3.84	29.90 ± 8.19	29.76 ± 8.34	8010	training-free
ZCNAS	90.21 ± 0.89	93.43 ± 0.64	70.71 ± 1.98	70.94 ± 1.93	42.09 ± 3.98	42.44 ± 4.06	12,915	training-free
TNASSE	89.99 ± 0.32	93.38 ± 0.35	70.39 ± 0.81	70.47 ± 0.76	42.95 ± 1.66	43.44 ± 1.71	549	training-free
NASWOT	89.69 ± 0.73	92.96 ± 0.81	69.86 ± 1.21	69.98 ± 1.22	43.95 ± 2.05	44.44 ± 2.10	306	training-free
TE-NAS	-	93.21 ± 0.68	-	70.21 ± 1.16	-	43.93 ± 2.14	1558	training-free
TEG-NAS	-	91.00 ± 0.33	-	70.10 ± 1.47	-	44.45 ± 0.75	9939.5	training-free
Ours	91.53 ± 0.24	94.27 ± 0.24	73.02 ± 0.82	73.19 ± 0.59	46.36 ± 0.73	46.70 ± 0.52	510	training-free
Ours-	90.83 ± 0.31	93.21 ± 0.38	71.29 ± 0.89	71.76 ± 0.78	44.58 ± 0.80	44.31 ± 0.84	472	training-free

**Table 2:** Experimental results compared on NAS-Bench-101.

Algorithm	CIFAR10		Cost
	**Valid**	**Test**	**GPUsec**
ResNet	90.83	93.97	-
AREA	93.67 ± 0.48	93.02 ± 0.48	12,000
REA	93.63 ± 0.50	93.02 ± 0.52	12,000
DARTs-V1	83.50 ± 0.11	82.74 ± 0.03	20,483
ENAS	93.83 ± 0.28	93.34 ± 0.26	22,325
NASWOT	92.47 ± 5.25	91.95 ± 5.18	140
ZCNAS	91.72 ± 1.80	91.29 ± 1.82	18,940
TEG-NAS	-	92.52 ± 1.30	2808
Ours	93.69 ± 0.57	93.01 ± 0.52	455
Ours-	92.27 ± 1.17	91.89 ± 1.13	401

**Table 3:** Statistical analysis of performance differences.

Comparison	*p*-Value	Effect Size (r)	Significance (α = 0.05)
Ours vs. NASWOT	<0.002	0.82	Yes
Ours vs. TEG-NAS	<0.002	0.71	Yes
Ours vs. REA	>0.3	0.58	No

**Table 4:** Ablation study on NAS-Bench-201 CIFAR10 with different indicators.

$S_{T}$	$S_{G}$	$S_{E}$	$S_{T}$ + $S_{G}$ + $S_{E}$
93.52 ± 0.31	92.73 ± 0.54	93.02 ± 0.41	94.27 ± 0.24

## Data Availability

All the datasets used in the paper are publicly available.

## Abbreviations

The following abbreviations are used in this manuscript:

NAS	Neural Architecture Search
RL	Reinforcement Learning
EA	Evolutionary Algorithm
BO	Bayesian Optimization
